# IL-22 Plays a Critical Role in Maintaining Epithelial Integrity During Pulmonary Infection

**DOI:** 10.3389/fimmu.2020.01160

**Published:** 2020-06-09

**Authors:** John F. Alcorn

**Affiliations:** Department of Pediatrics, UPMC Children's Hospital of Pittsburgh, Pittsburgh, PA, United States

**Keywords:** lung, interleukin, mucosa, influenza, bacteria

## Abstract

Pulmonary infection is a leading cause of hospitalization in world. Lung damage due to infection and host mediated pathology can have life threatening consequences. Factors that limit lung injury and/or promote epithelial barrier function and repair are highly desirable as immunomodulatory therapeutics. Over the last decade, interleukin−22 has been shown to have pulmonary epithelial protective functions at the mucosal immune interface with bacterial and viral pathogens. This article summarizes recent findings in this area and provides perspective regarding the role of IL-22 in mucosal host defense.

## Introduction

Protective immune mechanisms of lung injury during infection are an important area of research that could provide new immunomodulatory therapeutic options. Maintenance of lung epithelial structural integrity during bacterial or viral pneumonia is of critical importance to limit lung edema and pathogen dissemination. The function of the cytokine interleukin (IL)−22 in the lung has only recently been elucidated and much is still to be discovered. Work over the last decade has shown that IL-22 likely plays a role in epithelial biology during infectious diseases (1–4). IL-22 is a member of the IL-10 cytokine family and is produced mainly by innate and adaptive T cells in response to lung injury during infection, allergy, and fibrosis (5). IL-22 signals through a heterodimeric receptor of IL-22Ra1 and IL-10R2, which is expressed on non-hematopoietic cells. Ligand binding leads to phosphorylation of the transcription factor STAT3 and nuclear translocation to regulate transcriptomic program in cells. IL-22 signaling is opposed by a soluble decoy receptor IL-22Ra2, or IL-22 binding protein (IL-22BP), which is coordinately regulated with IL-22 production to regulate IL-22 signaling. In this perspective article, the role of IL-22 in regulating the epithelial barrier in the lung during bacterial, viral, and super-infection will be discussed.

## Bacterial Infection

Bacterial infections of the lung with Gram (−) and (+) bacteria are common causes of both community and hospital acquired pneumonia. The first description of a host defense role for IL-22 in the lung was demonstrated using the *Klebsiella pneumoniae* model of pneumonia (6). In that model, neutralization of IL-22 led to failed control of bacterial infection compared with control mice. Anti-IL-22 treatment resulted in increased bacterial burden in the lung and dissemination to the spleen. Induction of IL-22 in response to *K. pneumoniae* was dependent on IL-23 production. IL-23 is a canonical Type 17 immunity promoting cytokine. IL-22 promoted proliferation of human bronchial epithelial cells *in vitro* and improved transepithelial electrical resistance, a measure of barrier function, following scratch wounding. These data demonstrated an epithelial barrier protective effect of IL-22. Shortly thereafter, therapeutic delivery of IL-22 to rat lungs was shown to improve epithelial barrier function in a model of ventilator induced lung injury (7). IL-22 treatment reduced lung edema and increased survival in this model providing further evidence for IL-22 acting and an epithelial protective cytokine.

Using the *Pseudomonas aeruginosa* model of lung infection, antibody blockade of IL-22 was shown to increase infection, lung damage, and neutrophil accumulation (8). Consistent with this finding, exogenous IL-22 or neutralization of IL-22BP reduced neutrophil recruitment to the lung and pathology. More recently, IL-22–/– mice were tested in a model of *P. aeruginosa* infection (9). IL-22–/– mice had worsened lung injury when compared with wild-type (WT) control mice. These authors also showed that the inflammatory environment in the lung during *P. aeruginosa* infection results in proteolytic degradation of IL-22 via the neutrophil serine protease 3. Further, the *P. aeruginosa* protease IV has also been shown to degrade IL-22 as a potential virulence factor (10). These data suggest a critical role for IL-22 in Gram (−) host defense and identify targeting of IL-22 by pathogens as an immune evasion mechanism.

The role of the IL-22/IL-22BP axis in Gram (+) host defense has also been shown. Deletion or antibody neutralization of IL-22 resulted in increased lung bacterial burden and dissemination compared with controls during *Staphylococcus aureus* infection (11). These data are consistent with a barrier protective role for IL-22 in the lung. The role of IL-22 has also been studied in the context of *Streptococcus pneumoniae* infection (12, 13). IL-22–/– mice were shown to have increased bacterial burden in the lung compared to WT mice. Exogenous IL-22 was able to decrease *S. pneumoniae* burden in the lung in WT mice. In a second study, IL-22BP–/– mice, lacking the inhibitory receptor of IL-22, were protected from lung injury and mortality compared with WT mice. Further, IL-22BP–/– mouse lungs had altered transcriptomes with a lack of oxidative phosphorylation gene expression, which was IL-22 dependent. Macrophages from IL-22BP–/– mice had increased glycolytic activity, suggesting that IL-22 may regulate macrophage metabolism. While much about the mechanisms by which IL-22/IL-22BP regulate anti-bacterial host defense remains to be discovered, it is clear that IL-22 is of importance in these settings.

A potential relationship between IL-22 and interferon lambda (IFNλ) has recently emerged. IFNλ is an antiviral cytokine family that has been shown to be protective during viral infections without pro-inflammatory effects, an important consideration in the lung. In the *K. pneumoniae* model, IFNλR–/– mice exhibited better bacterial control and improved epithelial barrier function (14). These IFNλR–/–mice had acute elevations in IL-22 production suggesting that IFNλ negatively regulates IL-22 in the lung. This regulation may be bi-directional as IL-22–/– mice had decreased IFNλ expression in the later phases of infection. Using IL-22–/– mice, IL-22 was shown to be critical for epithelial barrier stability in the *K. pneumoniae* model, confirming aforementioned work using IL-22 neutralization. Consistent with the findings regarding IL-22 and IFNλ cross-talk, IL-22 treatment increased IFNλ production and anti-IL-22 decreased IFNλ levels in a model of *P. aeruginosa* infection and in an *in vitro* alveolar epithelial cell line (15). This interaction between IL-22 and IFNλ has also been suggested in models of bacterial pneumonia exacerbated by preceding influenza virus infection. IFNλR–/– mice were shown to produce increased levels of IL-22 following influenza, *S. aureus* super-infection (16). However, others did not observe an increase in IL-22 following exogenous IFNλ delivery in a similar super-infection model (17). These data suggest a potentially complex role for IL-22 in modulating antiviral immunity in the lung.

## Influenza Infection

Influenza virus is the cause of world-wide, annual epidemics and can result in severe lung pathogenesis in people of all ages. Initial study into the role of IL-22 during influenza infection produced limited results. Antibody neutralization of IL-22 was not effective at reducing influenza mediated morbidity or mortality in mice, although a reductive effect on viral load was shown (18). Shortly thereafter, influenza infection was shown to induce IL-22 production by invariant natural killer T (iNKT) cells in the lung (19). The pro-Type 17 cytokines IL-1β and IL-23 were shown to induce iNKT cell production of IL-22 and lead to protection from lung epithelial damage both *in vitro* and *in vivo*. In this study, IL-22 production was manipulated by deletion of iNKT cells and no effect on viral load was observed. These data suggest an epithelial protective role for IL-22 during viral infections.

These studies were then followed by a trio of studies of influenza infection in IL-22–/– mice. Conventional natural killer cells were shown to be a primary source of IL-22 following influenza challenge in mice (20). IL-22–/– mice had decreased epithelial regeneration compared to WT mice after influenza infection. In addition, IL-22 stimulated epithelial proliferation *in vitro*. Another study found similar effects on lung epithelial repair following influenza infection (21). In that study, IL-22–/– mice had increased lung injury, decreased lung function, and increased pulmonary fibrosis compared with WT mice 3 weeks following influenza infection. A third study provided additional supportive data showing increased lung injury and decreased epithelial integrity after influenza challenge (22). However, the primary cellular source of IL-22 was γδT and innate lymphoid cells in that study. All three studies failed to observe an impact on viral burden, but were able to show an important role for IL-22 in preservation of the epithelial barrier in viral pneumonia.

Regulation of IL-22 signaling during influenza infection has also been studied. Two studies showed that IL-22Ra1 is highly expressed on airway epithelial cells prior to influenza infection (20, 21). Following infection, expression of IL-22Ra1 is highly increased in injured areas of the distal lung. Tlr3 activation by viral pathogen-associated molecular pattern molecules in epithelial cells results in increased expression of IL-22Ra1 via IFNβ dependent STAT1 signaling (23). Additional pathways have been shown to regulate IL-22Ra1 protein stability. Glycogen synthase kinase 3β (GSK3β) was shown to phosphorylate IL-22Ra1 and increase its protein stability (24). Further, IL-22 was shown to inactivate GSK3β, perhaps as a feedback mechanism to terminate IL-22Ra1 signaling. IL-22Ra1 was also shown to be regulated via ubiquitination by the E3 ligase FBXW12 (25). This interaction destabilizes IL-22Ra1. During infections, neutrophil serine proteases also can degrade IL-22Ra1 providing another mechanism of control of IL-22 signaling (26). Regulation of IL-22Ra1 is complex and it is likely that we only have a limited understanding at this time.

Finally, deletion of IL-22BP negative regulation of IL-22 was shown to decrease lung inflammation and injury compared with WT mice after influenza infection (27). Increased tight junction protein expression was observed *in vivo*. Treatment of human bronchial epithelial cells with IL-22 resulted in increased expression of tight junction proteins and claudins, suggesting a direct role for IL-22 in mediating epithelial barrier integrity. Delivery of IL-22 to mice decreased influenza mediated inflammation and lung leak. IL-22 was also shown to inhibit viral induced expression of programmed death-ligand 1 (PD-L1) on epithelial cells (28). PD-L1 expression is a mechanism by which viruses evade T cell activation and clearance. In this manner, IL-22 may also promote antiviral immunity distinct from its barrier protective role. These data suggest that the IL-22/IL-22BP axis may be of critical importance in prevention of infectious lung injury.

## Influenza, Bacterial Super-Infection

An important clinical exacerbation of influenza infection is secondary bacterial pneumonia. In both epidemic and pandemic seasons, bacterial super-infection is a severe complication that leads to a need for intensive care and in some cases, death. *S. pneumoniae* has classically been the most common cause of influenza related super-infections, however over the last decade, *S. aureus* has become more predominant. IL-22–/– mice were shown to have increased bacterial burden and mortality compared with WT mice in a model of influenza, *S. pneumoniae* super-infection (22). In this model, exogenous IL-22 delivered to WT mice decreased bacterial dissemination, but did not affect lung bacterial load (29). IL-22 therapy increased epithelial barrier function and decreased lung leak. In support of these findings, deletion of IL-22BP in mice improved outcomes of influenza super-infection with both *S. aureus* and *S. pneumoniae* (30). Severe lung pathology induced by influenza, *S. pneumoniae* infection was significantly attenuated in IL-22BP–/– mice. Less lung leak and increased expression of claudin proteins were observed in IL-22BP–/– mice compared with WT mice. Finally, IL-22 treatment of air liquid interface cultures of human bronchial epithelial cells resulted in preserved tight junction function following injury with *S. aureus*. These data demonstrate and important role for IL-22/IL-22BP in the context of polymicrobial infection.

## Coronavirus Infection

The current COVID-19 pandemic has raised interest in immunomodulatory therapeutics. There are no published reports regarding lung IL-22 in coronavirus infections caused by severe acute respiratory syndrome viruses (SARS-CoV1 or SARS-CoV2) or middle east respiratory syndrome virus (MERS). A recent report suggests that IL-22 may be systemically elevated in the acute phase of SARS-CoV2 infection and may play a role in cardiovascular injury (31). Severe SARS-CoV2 infection is associated with development of acute respiratory distress syndrome (ARDS) with lung hyper-inflammation and edema (32, 33). The degree of similarity between SARS-CoV2 and influenza pathogenesis is currently unclear; however, it is likely that IL-22 plays a role in epithelial barrier integrity. Data is needed to assess the potential for targeting the IL-22 pathway in this context.

## Summary

The highlighted work and those of others have shown an emerging role for IL-22 in promoting epithelial integrity and repair following infectious pathogen challenge in the lung. Precise mechanisms of IL-22 action have been more elusive, as IL-22 often has limited biologic effects on uninjured cells *in vitro*. While data exist regarding the effects of IL-22 on epithelial cells *in vitro*, there remains much to be discovered. It will be important to assess the effects of IL-22 on epithelial cells in inflammatory settings, perhaps in combination with pathogen associated molecular patterns (PAMPs) or toxins. Evidence for direct impact of IL-22 on immune cells is less abundant and controversial. More work in this area is needed to determine if IL-22 affects lymphocyte function in the absence of signaling through lung stromal cells. Mechanisms by which IL-22 mediates pathogen clearance, in the case of bacterial infection, are mostly unknown. Several studies have shown that lung bacterial burden is altered when IL-22 is manipulated, indicating that IL-22's role is not solely focused on preventing dissemination from the lung. It is likely that IL-22, signaling through the epithelium, impacts host defense in several undiscovered ways. IL-22 has many described functions outside of the lung in the gastrointestinal tract, liver, and thymus. It is possible that IL-22 regulation of immunity in the lung is not restricted to direct effects in lung tissue. Focused use of floxed IL-22Ra1 mice should enable high resolution study of tissue and cellular compartments where IL-22 signaling is required during infection. Regardless, pre-clinical animal models suggest that IL-22 has significant therapeutic potential in the context of infectious diseases. Additional study is required to confirm the current reports in the literature and expand the field beyond the few pathogens mentioned herein. Undoubtedly, studies of IL-22 in SARS-CoV2 infection would be of great interest. Beyond pre-clinical animal models, translational studies are now needed to determine if IL-22 can limit lung damage and promote repair in humans. Less is known with regard to human IL-22 production and signaling in human infectious diseases. This area will be critical to evaluate in human pneumonia.

## Data Availability Statement

The original contributions presented in the study are included in the article/supplementary materials, further inquiries can be directed to the corresponding author/s.

## Author Contributions

JA conceived and wrote the manuscript.

## Conflict of Interest

The author declares that the research was conducted in the absence of any commercial or financial relationships that could be construed as a potential conflict of interest.
